# Pre-infestation of Tomato Plants by Aphids Modulates Transmission-Acquisition Relationship among Whiteflies, Tomato Yellow Leaf Curl Virus (TYLCV) and Plants

**DOI:** 10.3389/fpls.2017.01597

**Published:** 2017-09-22

**Authors:** Xiao L. Tan, Ju L. Chen, Giovanni Benelli, Nicolas Desneux, Xue Q. Yang, Tong X. Liu, Feng Ge

**Affiliations:** ^1^State Key Laboratory of Integrated Management of Pest Insects and Rodents, Institute of Zoology, Chinese Academy of Sciences Beijing, China; ^2^State Key Laboratory for Biology of Plant Diseases and Insect Pests, Institute of Plant Protection, Chinese Academy of Agricultural Sciences Beijing, China; ^3^State Key Laboratory of Crop Stress Biology for Arid Areas and the Key Laboratory of Crop Pest Management on the Losses Plateau of Ministry of Agriculture, Northwest A&F University Yangling, China; ^4^Department of Agriculture, Food and Environment, University of Pisa Pisa, Italy; ^5^INRA (French National Institute for Agricultural Research), UMR 1355-7254 Institute Sophia Agrobiotech, CNRS, Université Côte d’Azur Sophia Antipolis, France; ^6^Key Laboratory of Economical and Applied Entomology of Liaoning Province, College of Plant Protection, Shenyang Agricultural University Shenyang, China

**Keywords:** TaqMan Real-Time PCR, systemic induced defense, *Bemisia tabaci*, *Myzus persicae*, electrical penetration graph, jasmonic acid, salicylic acid, signaling pathways

## Abstract

Herbivory defense systems in plants are largely regulated by jasmonate-(JA) and salicylate-(SA) signaling pathways. Such defense mechanisms may impact insect feeding dynamic, may also affect the transmission-acquisition relationship among virus, plants and vectoring insects. In the context of the tomato – whitefly – Tomato Yellow Leaf Curl Virus (TYLCV) biological model, we tested the impact of pre-infesting plants with a non-vector insect (aphid *Myzus persicae*) on feeding dynamics of a vector insect (whitefly *Bemisia tabaci*) as well as virus transmission-acquisition. We showed that an aphid herbivory period of 0–48 h led to a transient systemic increase of virus concentration in the host plant (root, stem, and leaf), with the same pattern observed in whiteflies feeding on aphid-infested plants. We used real-time quantitative PCR to study the expression of key genes of the SA- and JA-signaling pathways, as well as electrical penetration graph (EPG) to characterize the impact of aphid pre-infestation on whitefly feeding during TYLCV transmission (whitefly to tomato) and acquisition (tomato to whitefly). The impact of the duration of aphid pre-infestation (0, 24, or 48 h) on phloem feeding by whitefly (E2) during the transmission phase was similar to that of global whitefly feeding behavior (E1, E2 and probing duration) during the acquisition phase. In addition, we observed that a longer phase of aphid pre-infestation prior to virus transmission by whitefly led to the up-regulation and down-regulation of SA- and JA-signaling pathway genes, respectively. These results demonstrated a significant impact of aphid pre-infestation on the tomato – whitefly – TYLCV system. Transmission and acquisition of TYLCV was positively correlated with feeding activity of *B. tabaci*, and both were mediated by the SA- and JA-pathways. TYLCV concentration during the transmission phases was modulated by up- and down-regulation of SA- and JA-pathways, respectively. The two pathways were inconsistent during the acquisition phase; SA- related genes were up-regulated, whereas those up- and down-stream of the JA pathway showed a more complex relationship. These findings enhance our understanding of plant – herbivore – virus interactions, which are potentially important for development of ecologically sound pest and pathogen management programs.

## Introduction

In recent decades, there have been many studies on the ecological and physiological mechanisms underlying plant-mediated interactions among herbivore arthropods and plant pathogens, particularly regarding plant defense responses (Al-Bitar and Luisoni, 1995; Beale et al., 2006; Bleeker et al., 2011; Baldin et al., 2013; Mouttet et al., 2013). Although it is well established that infection by pathogenic microorganisms may indirectly influence the behavior of the subsequent herbivore insects of the plant (Beale et al., 2006; Goggin, 2007; Mouttet et al., 2011), there has been less work on the effect of insect herbivore infestation on plant pathogens (Czosnek et al., 1988; Mouttet et al., 2011, 2013). The defense responses induced in plants after incidence of herbivory is central to the longstanding co-evolutionary system of host plant, pathogenic microorganism and herbivore arthropod (including vectors). Furthermore, these responses often impact prevalence of disease and pests, as well as the degree of damage to plants (Mayer et al., 2002; Cory and Hoover, 2006; Huot et al., 2013). However, there are key gaps in understanding of host plant – vector insect *–* pathogen interaction systems. For example, it is unclear how phytohormone-mediated plant defenses which are induced by insect infestations or virus transmission may influence behaviors of virus-carrying insects. Thus, understanding the mechanisms by which virus transmission occurs in plants showing herbivore-induced defense may be helpful for development of novel pest management strategies.

There have been numerous studies describing various defense systems that are induced in plants in response to damage caused by pathogens and herbivore arthropods (Karban and Baldwin, 1997; Huot et al., 2013). Key signaling pathways identified as regulators of plant defense are Jamonate (JA) for insect infestations and Salicylate (SA) for pathogen infestations, although the relationship between gene expression and function can be complex (Thaler et al., 2010). Further, understanding signal interactions within a plant – insect – pathogen system requires consideration of ecological context and the temporal sequence of interactions, such as the effect that an initial herbivore attack might have on the system during subsequent infestations. These types of interactions are more representative of incidents in natural and agricultural settings, and thus must be studied to develop more effective solutions for pest/pathogen management (Agrawal et al., 2000; Howe and Jander, 2008; Taggar and Gill, 2016).

Tomato Yellow Leaf Curl Virus is a cosmopolitan geminivirus which can infect dicotyledons, including tomato (Goggin, 2007; Heil, 2008), and is usually transmitted by the globally invasive whitefly *Bemisia tabaci* (Green and Ryan, 1972; Rubinstein and Czosnek, 1997). The transmission of TYLCV by *B. tabaci* causes huge losses in tomato yield in many regions, including China (Pan et al., 2012). The acquisition of TYLCV by *B. tabaci* occurs when a non-infected whitefly feeds on the phloem of a TYLCV-infected plant, and transmission occurs when a virus infected *B. tabaci* feeds on a previously healthy plant (Czosnek et al., 2002). It has been shown that within this defense system, a prior attack by aphid *Myzus persicae* can influence the feeding strategy and population dynamics of subsequent herbivores (Tan et al., 2014). It is unclear whether TYLCV transmission and acquisition are also affected by the feeding behavior of whiteflies, or whether these are influenced by aphid pre-infestation.

Plants infected by TYLCV may attract more whiteflies, and generally create conditions suiting colonization and suppression of the virus (Stout et al., 2006; Luan et al., 2013). Previous research has shown that plant defense responses induced by aphids are similar to those induced by pathogens (Thompson and Goggin, 2006). Aphid attacks can activate the SA-signaling pathway and suppress the JA-signaling pathway of the plant, and consequentially attract more whiteflies (via JA) and suppress TYLCV (SA) (Zarate et al., 2007; Verhage et al., 2010). Increasing this attraction can lead to aggregation of whiteflies and increase their feeding, both of which may increase TYLCV transmission and acquisition. Therefore, evaluation of the SA- and JA-pathway under aphid pre-feeding may help us to understand the mechanism by which induced defense is regulated.

In order to better understand the interactions in the plant- herbivore-virus system under conditions of sequential herbivory, we tested the hypothesis that pre-infestation by aphid *M. persicae* affects the expression of hormone-related genes and the SA- and JA-signaling pathways, consequentially impacting feeding behavior of subsequent whiteflies and thus the transmission and acquisition of TYLCV. TaqMan Real-Time PCR has been used for rapid and efficient quantitative detection of organismal DNA. It has proven highly sensitive for detecting DNA viruses in insect vectors and plants (Lei et al., 1987; Kunkel and Brooks, 2002), and there have been reports on using this technology for detection of TYLCV in tomato (Liu et al., 2007). We use Real-Time PCR to quantify TYLCV in infected tissues of tomato plant as well as in *B. tabaci*. We tested if pre-infestation with *M. persicae* (i) affect the feeding behavior of *B. tabaci*, (ii) modulate TYLCV transmission and acquisition between host plant and *B. tabaci*, and (iii) impact the expression of SA- and JA-signaling pathway genes (and potential link with vector feeding behavior and virus transmission and acquisition).

## Materials and Methods

### Virus, Insects, and Plants

Tomato plants, *Solanum lycopersicum* L. (var. Baofen-F1, 2008, Changfeng Institute of Vegetable, Lintong, Xi’an, Shaanxi, China) were cultivated in plastic trays (50.0 × 25.0 × 15.0 cm), with eight plants per tray. Seedlings, about 4–5 cm in height, were transplanted into plastic pots (20 cm in depth and 15 cm in diameter) and placed in clean cages (60 × 60 × 60 cm, plastic frame, screened with 120-mesh nylon yarn net). Plants used in all experiments were approximately 30 cm in height with 4–6 true, fully expanded leaves. The experiments were conducted in an environmental chamber at 25 ± 2°C, 65 ± 5% relative humidity and photoperiod of 16/8 h of light/dark with artificial lighting of 3500 lux. About 15,000 *M. persicae* individuals were collected from pepper plants (*Capsicum annuum* L. var. Jingyuan New Prince, provided by Qing County Modern Agricultural Technology Promotion Center, Hebei Province) in a greenhouse on the campus of Northwest A&F University, Yangling, Shaanxi (116°22′42″E and 39°59′58″N) in March 2011. The aphids were maintained on tomato plants under the laboratory conditions described above. All tested aphids were offspring (>five generations) of the original collected specimens.

*Bemisia tabaci* whiteflies (507 males and 631 females) of Middle East-Asia Minor 1 (MEAM 1) species were collected from tomato plants in a greenhouse and their identity confirmed by mitochondrial DNA marker analysis as described by Chu et al. (2007). They were reared on cotton plants under the same conditions described for green peach aphids during March and April 2011. Newly emerged adult whiteflies were used in all experiments after they had occupied the tomato plants for more than five generations. TYLCV-infected tomato plants were provided by the Natural Enemy Application and Research Laboratory, Institute of Plant and Environment Protection, Beijing Academy of Agriculture and Forestry Sciences, Beijing, China. The plants were cultivated in the State Key Laboratory of Crop Stress Biology of Northwest A&F University using ventilated culturing cages (60 × 60 × 60 cm, plastic frame, screened with 120-mesh nylon yarn net) containing 100 pairs of whitefly adults. The whiteflies were moved to the cages containing 15 tomato plants with 4–6 leaves each. TYLCV-infected plants were tested after 20 days. *Bemisia tabaci* specimens were obtained from cotton leaves from which pseudo pupae had been cut off and the petiole had been inserted in a glass bottle filled with water and placed in a plastic cage (13 × 13 × 30 cm; 100-mesh nylon yarn net door on one side). Non-viruliferous whiteflies were maintained on cotton plants (*Gossypium arboreum* cv. SN407) that had been identified as non-infected hosts for TYLCV. Newly emerged Non-viruliferous adult whiteflies were used in experiments after they were moved from cotton plants and had occupied the tomato plants for more than five generations. Newly emerged whiteflies were collected and released onto the TYLCV-infected tomato plants in ventilated culturing cages for a duration of 48 h, after which the whiteflies were used in the experiments; previous studies indicate that the viral concentration in whitefly peaks after 12–48 h of feeding on tomato plants infected with TYLCV (Mason et al., 2008; Luo et al., 2010; Lucas-Barbosa et al., 2011).

### DNA Extraction and Virus Detection

Leaves of five TYLCV-infected tomato plants were ground into powder in a pre-chilled motor in liquid nitrogen. Genomic DNA was extracted from 40 mg powder using the Wizard^TM^ Genomic DNA purification kit (Promega Corporation, Madison, WI, United States) according to manufacturer’s instructions. Genomic DNA from healthy leaves of non-infected tomato plants were used as control. The extracted DNA was used as a template for PCR reaction or kept at -20°C for later use.

Based on the complete genomic sequence of TYLCV (GenBank accession number: NC_004005.1), two primer pairs: F1 (5′-CCAATAAGGCGTAAGCGTGTAGAC-3′) and R1 (5′-ACGCATGCCTCTAATCCAGTGTA-3′), F2 (5′-TCCCCTCTATTTCAAGATAACAGAACA-3′) and R2 (5′-TCTGAGGCTGTAATGTCGTCCA-3′), and TaqMan probe (5′-FAM- CCCAATGCCTTCCTG-MGB-3′) were designed by GeneCore Bio Technologies Co. Ltd. (Shanghai, China) to amplify 543 and 161 bp regions of the TYLCV gene. Actin (ACT) gene of *B. tabaci* (GenBank accession number: AF071908) and S. lycopersicum (GenBank accession number: AB199316) were used as internal controls, and 174 and 191 bp regions of BtACT and SlACT genes were amplified using F3 (5′-TCCTTCCAGCCATCCTTCTTG-3′) and R3 (5′-CGGTGATTTCCTTCTGCATT-3′) (Li et al., 2013) and F4 (5′-TGGTCGGAATGGGACAGAAG-3′) and R4 (5′-CTCAGTCAGGAGAACAGGGT-3′) (Chen et al., 2017) as primer pairs, respectively.

**FIGURE 1:**
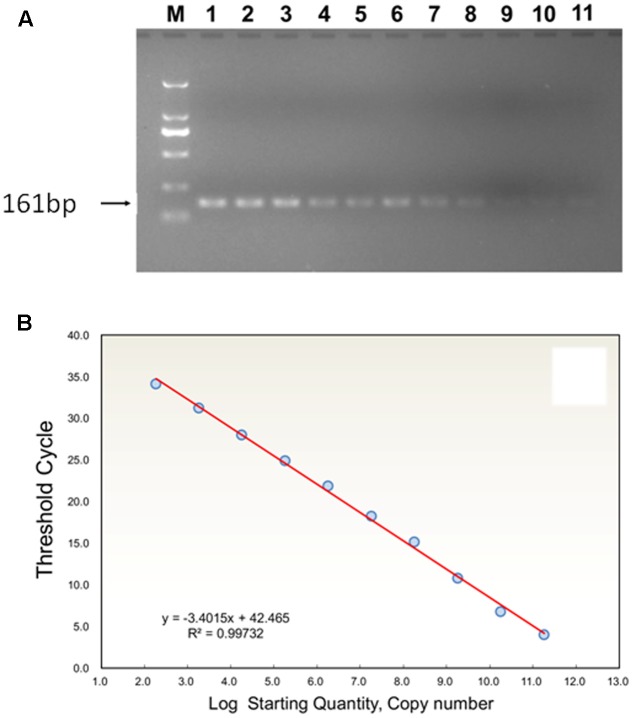
**(A)** Agarose gel analysis of TYLCV (TYLCV means tomato yellow leaf curl virus) using serial dilutions of plasmid standard product DNA, showing the amplification products obtained by PCR with TYLCV; M: 161-bp ladder DNA marker. Standards (copy numbers per μl of target DNA): 1, 9.16 × 10^10^; 2, 9.16 × 10^9^; 3, 9.16 × 10^8^; 4,9.16 × 10^7^; 5, 9.16 × 10^6^; 6, 9.16 × 10^5^; 7, 9.16 × 10^4^; 8, 9.16 × 10^3^; 9, 9.16 × 10^2^; 10, 9.16 × 10^1^; 11, 9.16 × 10^0^. **(B)** Standard curve analysis: the relationship between TYLCV concentration and corresponding Ct value is shown by linear regression equation of Ct (x) transformed into log (starting quantity).

The PCR reactions were conducted in a C1000 Thermal Cycler (BioRad, Hercules, CA, United States) using F1 and R1 as primer pair in 30 μL of mixture containing 1 μL of template DNA, 1 μL of each primer (10 μM), 3 μL of 10 × PCR buffer (150 mM Tris–HCl, 500 mM Tris–HCl, pH 8.0 with 25 mM MgCl_2_), 1 μL of dNTP Mix (2.5 mM), 0.5 μL of Premix *Ex* Taq DNA polymerase (5 U/μL, Takara Biotechnology Co. Ltd., Dalian, China) and 22.5 μL of double distilled H_2_O. The PCR conditions were 10 min at 94°C, followed by 40 cycles of 1 min at 94°C, 1 min at 57°C and 2 min at 72°C and a final extension at 72°C for 10 min. The PCR products were separated on a 2% agarose gel and purified using the Biospin Gel Extraction Kit (Bioer Technology Co. Ltd., Hangzhou, China) as described by the manufacturer. The recycled PCR products were ligated into the pMD-19 T vector (Takara) and transformed into DH5α cells. After blue-white selection, positive clones were sequenced by the Shanghai Sunny Biotech Co. Ltd., China. The sequence-verified clones were cultivated and plasmids were extracted. The concentration of extracted plasmid DNA was measured using a UV-Vis spectrophotometer (NanoDrop 2000 c). Copy number was determined according to the equation: copy number/mL = 6.02 × 10^23^ (cn/mol) × plasmid concentration (g/mL)/MW (g/mol). The standard curve of TYLCV coat protein gene was obtained by using serial 10-fold diluted plasmids (9.16 × 10^10^ to 9.16 × 10 copies) as templates, and by using F2 and R2 as primer pairs and TaqMan fluorescence probe (**Figure 1**). Amplifications were performed in 25 μl volumes containing 12.5 μL of Premix Ex Taq (Probe qPCR), 2 μL of template, 0.5 μL of each 10 μM F2 and R2 primers using thermocycler conditions as follows: 95°C for 10 s, followed by 40 cycles of 10 s at 95°C and 30 s at 58°C. Then, the linear equation of log concentration versus Ct curve was generated, where Ct values were plotted from 10-fold serial dilutions of the plasmid DNA. The estimation of copy number in samples was performed by computing the estimates of linear regression coefficients. The quantifications of DNA samples were calculated based on the fluorescence (ΔRn) values. All samples were run in duplicate by TaqMan Real-Time PCR assay for accuracy of results.

High specificity was determined for primers (**Figure 1**). PCR amplification showed that the product of TYLCV DNA corresponded to a 161 bp band, and lacking primer dimers. The data for the estimation of a standard curve for absolute quantitative and TaqMan Real-Time PCR assays were obtained and analyzed by an iQTM5 Multicolor Real-Time PCR Detection System. Thus, sample Ct values were obtained automatically. Ct values and the logarithm of TYLCV DNA copy number (log) had a linear relationship. The logarithmic average of TYLCV DNA copy number (log) was calculated through a standard curve, giving y = -3.4015x + 42.465, slope -3.4015, *R*^2^ = 0.9973 from each sample of TYLCV, an amplification efficiency (E) of 96.8%, and showed a robust linear relationship (**Figure 1B**).

### Impact of Aphid Pre-infestation Duration on TYLCV Transmission and Acquisition by Whiteflies on Tomato Plants

We assessed pre-infestation effects on viruses, separately for the processes of transmission and acquisition, and each by varying the duration of pre-infestation. We sequentially added aphids then whiteflies, then measured virus concentration. For transmission, Each tomato plant with 4–5 expanded leaves was infested separately with 60 fourth-instar aphid nymphs, then the aphids were removed using a soft brush after 12, 24, or 48 h. Aphids were randomly assigned to each plant. Sixty viruliferous adult whiteflies were introduced throughout the tomato plant (with sufficient space for flight in the mini cage) and fed for 24 h after removal of aphids. The whiteflies were removed using an aspirator. A group of five tomato plants (including roots) was collected, the roots washed with sterile distilled water, and stored at -80°C. Muniyappa et al. (2000) showed that the virus can be transmitted from whiteflies that have fed on TYLCV-infected plants for 24 h, to uninfected plants after exposure of only 20 min. Jiu et al. (2006) reported that the shortest time of transmission of TYLCV to plants by whiteflies was 15–30 min. Thus, in the present study, a 24 h feeding time was thought sufficient to transmit TYLCV to tomato plants. The experiment was repeated five times, each time with five tomato plants. Control tomato plants were not infested with aphids. Leaves of five TYLCV-infected tomato plants were ground into powder in a pre-chilled motor in liquid nitrogen. Virus concentration was assessed by DNA extraction and PCR; the TaqMan PCR amplification system followed the methods described above. For virus acquisition, we also measured TYLCV for *B. tabaci* via feeding on virus-infested tomato plants. Viruliferous whitefly were first used to transmit virus to the tomato plant, followed by aphid preinfestation and subsequent whitefly exposure. Tomato plants treated as above were individually placed in a square plastic cage (13 × 13 × 30 cm), and 60 viruliferous adult whitefly introduced using an aspirator. The whitefly adults were removed after feeding for 24 h, and then the plants were pre-infested with aphids as above. Fifty newly emerged non-viruliferous whitefly adult females were introduced into the cage to acquire virus from the infected tomato plant after removing the aphids. After feeding for 24 h, 40 whiteflies per plant from each group of five tomato plants were collected and stored at -80°C. Control tomato plants were not infested with aphids. The experiment was replicated five times, each time with five different tomato plants. For virus detection in whitefly, DNA was extracted using the Wizard^TM^ Genomic DNA purification kit (Promega, Madison, WI, United States) from 200 whiteflies of a group of five tomato plants, which were ground into homogenate in 600 μL of nuclear pyrolysis liquid. The extracted DNA was diluted 10 times for TaqMan PCR assay or saved at -28°C for later use. PCR amplification system and response procedures were as reported above.

### Effects of Whitefly Feeding Behavior on Virus Transmission and Acquisition

#### Tomato Plants Were Treated as Outlined in Section “Impact of Aphid Pre-infestation Duration on TYLCV Transmission and Acquisition by Whiteflies on Tomato Plants”

Each tomato plant with 4–5 expanded leaves was infested separately with 60 fourth-instar aphid nymphs, then the aphids were removed using a soft brush after 24 or 48 h. Aphids were randomly assigned to each plant. Because the concentration of TYLCV was similar for aphid feeding of 12 and 24 h, this test used tomato plants infested with aphids for 24 or 48 h. The experiment was repeated three times, each time with eight whiteflies recorded.

For transmission, whitefly feeding during the virus transmission phase was recorded. Feeding behavior was assessed for newly emerged viruliferous whiteflies from TYCLV infected tomato plants. For acquisition, whitefly probing behavior during virus acquisition (from tomato) was recorded for newly emerged whiteflies from non-infected tomato.

Whitefly feeding was monitored using the electrical penetration graph (EPG) method (Liu et al., 2013). In EPG, the insect and its host plant creates an electrical circuit that is completed when the mouthparts of the phloem-feeding insect penetrate the plant. The resulting electrical signals are amplified and digitized. Fluctuations in voltage and electrical resistance are recorded on computer, and can be matched to specific feeding events. Prior to recording, whiteflies were immobilized by placing them in a freezer for several minutes, then waiting 1 h without feeding. The recording electrode of the EPG system (a thin gold wire of length 2 cm and diameter 10 μm), was connected to the whitefly’s dorsum with silver paint. Electrode adhesion was conducted under a microscope, and then the whitefly was put on the abaxial surface of the leaf and allowed to crawl freely. The wired whiteflies were then connected to the Giga-8 probe input, and another electrode was inserted into the soil at the base of the tomato plant. The whole system was placed into an electrically grounded Faraday cage to guard against external electrical noise. The EPG signals were digitized with a DI710-UL analog-to-digital converter, and the output was acquired and stored with PROBE3.4 software. The data were analyzed subsequently with STYLET 2.0 software. Phases of feeding behavior are described by 14 EPG parameters related to the pathway phase (C waveform, F waveform and potential drops), phloem phase (E waveform) and xylem phase (G waveform). Phloem subphase 1 (E1: salivary secretion into sieve elements) and phloem subphase 2 (E2: phloem ingestion) were extracted from each recording and compared among treatments. 12 h of EPGs were continuously recorded for each replicate. All experiments were carried out under artificial light (1,500 lx) with a 16 h light/8 h dark regime and at 25°C ± 2°C, at 70% relative humidity (RH).

### Aphid Pre-infestation Effects on Tomato Defense-Related Genes before Transmission and Acquisition

Each treatment combination was replicated three times with eight plants for biological replicates, and each biological replicate contained three technical replicates. The expression of target genes involved in the JA-signaling [*lipoxygenase D* (*LOXD*), *proteinase inhibitor II* (*PI-II*)] and SA-signaling [*phenylalanine ammonia lyase* (*PAL*), and *β-1,3-glucanase* (*PR2b*)] pathways were determined.

The RNeasy Mini Kit (Qiagen, Valencia, CA, United States) was used to isolate total RNAs from tomato leaves (100-mg samples) before viruliferous whitefly and non-infected whitefly feeding, and 1 μg of RNAs were used to generate the cDNAs. The mRNA amounts of seven target genes were measured by real-time quantitative PCR.

Specific primers for each gene were designed from the tomato plant expressed sequence tag sequences using PRIMER5 software (**Table 1**). The reaction mixture contained the following reagents: 1 μL of first-strand cDNA, 2 μL of reaction buffer, 1 μL of MgCl_2_ (50 mM), 0.3 μL of SYBR Green, 0.2 μL of PLATINUM Taq polymerase, 0.5 μL of dNTPs (10 mM) and 10 μM of each primer in a total volume of 20 μL. Reactions were carried out on the Mx 3500P detection system (Stratagene), and PCR was performed under the following conditions: 2 min at 95°C; followed by 40 cycles of 30 s at 95°C and 30 s at 60°C. Following the real-time quantitative PCR, a melting curve was generated by gradually increasing the temperature to 95°C to test the homogeneity of PCR products. Relative standard curves for the transcripts of every gene were prepared with linear gradient (five-fold) cDNA as template and were included within each real-time quantitative PCR. The relative level of each target gene was standardized by comparing the copy numbers of target mRNA with *SlACT* and *SlTUB* CT values, which remained constant under different treatment conditions. The fold-changes of target genes were calculated using the 2^ΔΔC_T_^ method. All PCR runs were performed with three technical replicates. The three independent biological replicates, each containing eight plants, were analyzed.

**Table 1:** Overview of the target genes used in this study, showing their GenBank accession numbers and the primer pair used for qRT-PCR.

Target gene	GenBank no.	Sequence (5′-3′)	Length (bp)
*SlTUB-F*	DQ205342	AACCTCCATTCAGGAGATGTTTT	180
*SlTUB-R*		TCTGCTGTAGCATCCTGGTATT	
*SlActin-*F	AB199316	5′-TGGTCGGAATGGGACAGAAG-3′	191
*SlActin*-R		5′-CTCAGTCAGGAGAACAGGGT-3′	
PAL-F	M83314.1	5′-ACAGAATTGTTGACGGGTGA-3′	122
PAL-R		5′-CCATTCCAGCTCTTCAGACA-3′	
PR-2b-F	M80608.1	5′-CCCATTTCAAGTTCCTGCTT-3′	112
PR-2b-R		5′-AGAATTGCCAATCAACGTCA-3′	
LOXD-F	U37840.1	5′-GGCTTGCTTTACTCCTGGTC-3′	72
LOXD-R		5′-AAATCAAAGCGCCAGTTCTT-3′	
*PI -II* -F	AY129402.1	5′-TGATGAACCCAAGGCAAATA-3′	154

**^∗^SlTUB* and *SlActin* are reference genes*.

### Data Analysis

One-way analysis of variance (ANOVA) was used to analyze the data of log copy number of TYLCV DNA for the effects of duration of infestation by *M. persicae* on the transmission and acquisition of TYLCV, whitefly probing profiles (EPG) and defense gene expressions. ANOVA was performed with IBM SPSS statistics version 20.0 (SPSS, Chicago, IL, United States). An independent *t*-test was used to test the effects of aphid feeding duration on defense gene expression during transmission and acquisition. All data were checked for normality and equality of residual error variances, and were transformed (log or square root) where appropriate to satisfy assumptions of ANOVA. General Linear Mixed Model (GLMM) was used to analyze the effects of the duration of aphid pre-infestation, plant position and their interaction on TYLCV. In both cases, when a significant effect was found, Duncan’s test was used as *post hoc* test to compare the log copy number of TYLCV DNA (*P <* 0.05).

## Results

### Impact of Aphid Pre-infestation Duration on TYLCV Transmission and Acquisition by Whiteflies

**FIGURE 2:**
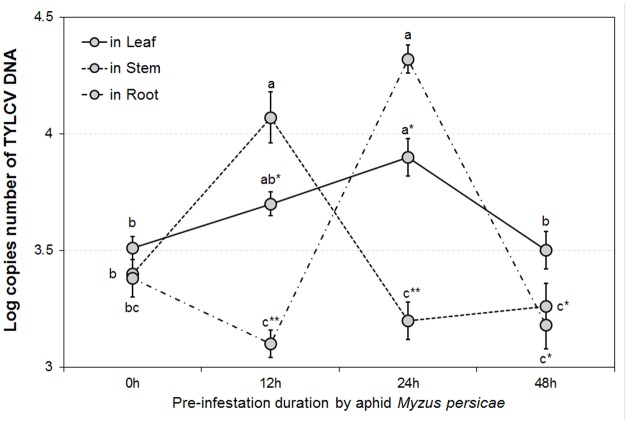
TYLCV DNA concentrations in different tomato plant parts after feeding by TYLCV-infested *Bemisia tabaci* according to various *Myzus persicae* pre-infestation duration treatments. The error bars are standard errors of the mean. Colored letters indicate significant differences of DNA concentration among different aphid pre-infestation durations in each tomato plant part, and ‘^∗^’ indicates significant differences among three plant parts in the same pre-infestation duration according to Duncan’s test at *P* = 0.05.

**FIGURE 3:**
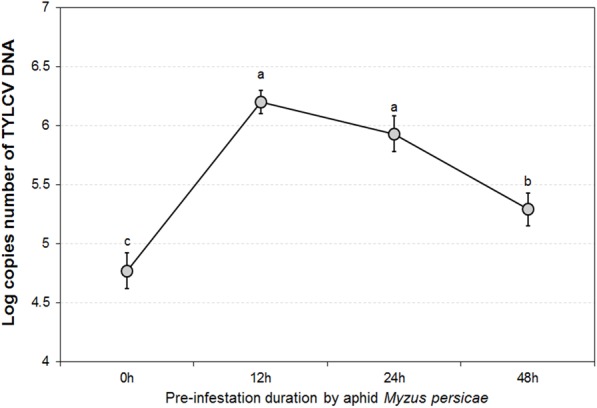
TYLCV DNA concentrations in *Bemisia tabaci* adults after feeding on TYLCV-infested tomato plants for 24 h according to various *Myzus persicae* pre-infestation. The error bars are standard errors of the mean. Letters indicate significant differences of DNA concentration among different aphid pre-infestation durations according to Duncan’s test at *P* = 0.05.

The GLMM tests highlighted significant differences in TYLCV DNA concentrations for different durations of aphid pre-infestation (*F* = 12.622, *df* = 3176, *P* < 0.05), and among different tomato plant tissues (*F* = 4.107, *df* = 2177, *P* < 0.05). Further, there was a significant interaction between duration of aphid pre-infestation, tomato plant tissues, and concentration of TYLCV DNA (*F* = 7.208, *df* = 6, *P* < 0.05) (**Figure 2**). The concentration of TYLCV DNA in *B. tabaci* after feeding on virus-infected tomato was influenced by duration of aphid pre-infestation (*F* = 3.769, *df* = 3176, *P* < 0.05; **Figure 3**).

### Aphid Pre-infestation Duration Effects on Feeding Behavior of Whiteflies during Transmission and Acquisition Phase

#### Transmission

**FIGURE 4:**
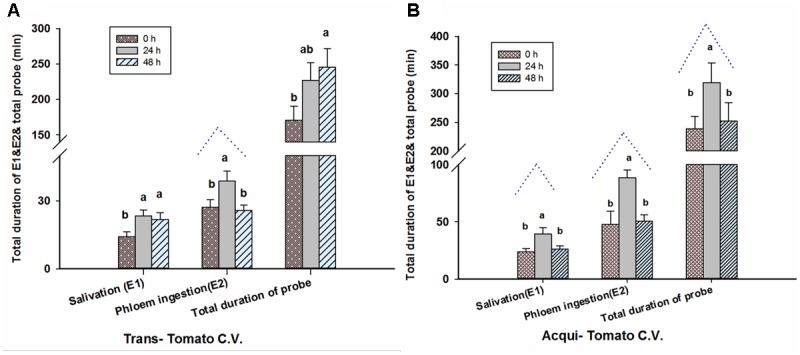
Aphid pre-infestation duration effects on feeding behavior (EPG) of transmission **(A)** of whiteflies on virus non-infected tomato plants and acquisition **(B)** of whiteflies on virus infected tomato plants. Letters indicate significant differences of feeding behavior among different aphid pre-infestation durations.

Twenty-four hour aphid infestation significantly increased the time that viruliferous whiteflies spent in the salivation phase [E1] (*F* = 3.586, *P* = 0.033, *df* = 2, 69) and duration of phloem feeding [E2] (*F* = 4.296, *P* = 0.017, *df* = 2, 69; **Figure 4A**). By contrast there was no significant effect on the total duration of probing by whiteflies (*F* = 2.658, *P* = 0.077, *df* = 2, 69; **Figure 4A**). For 48-h aphid infestation, the duration of E2 was similar to plants not infested with aphids; whereas significant differences are observed at 48 h for the duration of E1 and total duration of probing, compared to 0 h.

#### Acquisition

Pre-infestation with *M. persicae* significantly impacted several aspects of whitefly feeding on virus-infected leaves (E1: *F* = 4.836, *P* = 0.011; E2: *F* = 6.173, *P* = 0.003; and total duration of probing: *F* = 3.669, *P* = 0.031; all *df* = 2, 69; **Figure 4B**). 24 h of aphid infestation significantly increased E1, E2 and the total duration of probing by whiteflies. For 48-h aphid infestation, the durations of E1, E2 and of probing were similar to plants not infested with aphids. This trend was similar to the virus concentration in whiteflies with increasing aphid feeding duration on viruliferous tomato plants (**Figure 4B**).

### Effects of Aphid Pre-infestation on Defense Genes in Tomato Prior to Transmission and Acquisition

**FIGURE 5:**
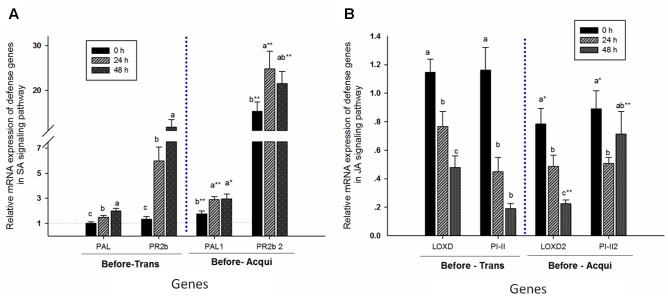
Aphid pre-infestation duration effects on related defense genes in the SA- **(A)** and JA- **(B)** signaling pathways in tomato plants. The relative mRNA expression was calculated according to the 2^-ΔΔC_T_^ method, and the *y* axis represents the level of change. The expression level of each target gene was first normalized to the quantity of *SlActin* and *SlTub*. The error bar represents the standard deviation calculated from three replicates. Letters indicate significant differences in relative expression among different aphid pre-infestation durations, and ‘^∗^’ indicates significant differences in the same aphid pre-infestation duration according to Duncan’s test at *P* = 0.05.

The relative mRNA expressions of *PAL* (before transmission: *F* = 9.213, *P* = 0.0001, *df* = 2, 69; and before acquisition: *F* = 4.727, *P* = 0.012, *df* = 2, 69) and *PR2b* (before transmission: *F* = 19.881, *P* = 0.0001, *df* = 2, 69; and before acquisition: *F* = 2.553, *P* = 0.085, *df* = 2, 69) positively correlated with aphid feeding time prior to transmission by whiteflies (**Figure 5**). Similarly, prior to acquisition by whiteflies, expressions of *PAL* and *PR2b* in plants with 24 h of aphid feeding were higher than for those without aphids, but similar to those with 48 h of aphid feeding. Moreover, expressions of both genes were higher prior to acquisition by whiteflies than prior to transmission by whiteflies: *PAL* for 0 h, *t* = -2.924, *df* = 33.022, *P* = 0.006; for 24 h, *t* = -4.709, *df* = 35.63, *P* = 0.0001; and for 48 h, *t* = -2.132, *df* = 35.117, *P* = 0.04; and *PR2b* for 0 h, *t* = -6.654, *df* = 23.494, *P* = 0.0001; for 24 h, *t* = -4.547, *df* = 26.518, *P* = 0.0001; and for 48 h, *t* = -3.111, *df* = 35.117, *P* = 0.003 (**Figure 5**).

In contrast, the relative mRNA expressions of JA-signaling genes *LOXD* (before transmission: *F* = 12.418, *P* = 0.00001, *df* = 2, 69; and before acquisition: *F* = 12.566, *P* = 0.00001, *df* = 2, 69) and PI-II (before transmission: *F* = 20.617, *P* = 0.00001, *df* = 2, 69; and before acquisition: *F* = 2.611, *P* = 0.081, *df* = 2, 69) were significantly reduced with increase in aphid feeding time, except for *PI-II* in plant prior to acquisition by whiteflies. The expression of *LOXD* in plants was lower prior to acquisition than prior to transmission by whiteflies, except for during an aphid infestation period of 24 h (*LOXD* for 0 h, *t* = 2.497, *df* = 46, *P* = 0.016; for 24 h, *t* = 1.666, *df* = 45.831, *P* = 0.103; and for 48 h, *t* = -2.741, *df* = 28.652, *P* = 0.01). However, expression of *PI-II* was lower in the non-infested plant prior to acquisition but not prior to transmission (*PI-II*, 0 h: *t* = 2.304, *df* = 46, *P* = 0.026; 24 h: *t* = -0.532, *df* = 30.834, *P* = 0.598; 48 h: *t* = -4.889, *df* = 27.284, *P* = 0.00001) (**Figure 5**).

## Discussion

This study is the first investigation on impact of herbivore pre-infestation on virus transmission and acquisition by subsequent herbivores, in terms of key interactions such as feeding behavior and plant defenses. We demonstrated that virus transmission and acquisition between tomatoes and vector whiteflies under aphid pre-infestation follow two different mechanisms. The SA- and JA-signaling pathways had different roles and the feeding behavior of whiteflies dominated the acquisition process. We believe that this overlooked issue will be of wide ecological interest, since knowledge of the effects of herbivorous feeding activity on the acquisition and transmission of viruses can shed light on complex plant-mediated relationships among herbivores, arthropod vectors and pathogens (Mehta et al., 1994; Messina et al., 2002; Beale et al., 2006; Mithöfer and Boland, 2012). Feeding behavior (salivation/phloem ingestion/duration of feeding) of viruliferous and non-infected *B. tabaci* was also influenced by different aphid pre-infestation times, in contexts of both transmission and acquisition. Furthermore, we found that the expression of two SA-signaling pathway genes may increase when aphid pre-infestation period is prolonged, and expression of the related JA-signaling pathway is reduced, especially during the transmission phase. Previous studies have shown that attack by aphids can inhibit the JA-signaling pathways and consequentially reduce negative impacts of herbivory (Papayiannis et al., 2009, 2010; Giordanengo et al., 2010). Plant defense responses induced by sap-sucking herbivores or pathogens are often similar, since both responses can act to resist both insects and pathogens (Thaler et al., 2002; Murugan and Dhandapani, 2007). The defense signaling pathways relying on SA and JA may vary with injury time (Preston et al., 1999; Nombela et al., 2009). Tobacco plants infected with TMV developed SAR, while damage response via JA was not normally activated, possibly because infection by TMV increased SA levels, inhibiting the JA pathway (Ohgushi, 2005). However, this might reduce JA/ET-associated insect resistance (Salzman et al., 2005).

### TYLCV Transmission/Acquisition

The pattern in transmission of virus from viruliferous *B. tabaci* to non-infected tomato was consistent among different plant organs tested (attacked leaves, stems, and roots), as well as under different durations of aphid pre-infestation. When measured directly on the aphid-attacked leaf, the amount of TYLCV initially increased, and was followed by a decline, with extension of period of pre-infestation. The stem and the root showed similar tendencies; the pre-infestation was found to stimulate systemic resistance, resulting in a more effective response to subsequent attacks. Generally, herbivore feeding action leads to a diverse and synergistic systemic defense, performed across different tissues of the plant (Pico et al., 1999; Schilmiller and Howe, 2005; Jung et al., 2009). The defense pathways may be induced in tissues away from the position of attack, although this reaction is somewhat delayed (Kessmann et al., 1994). In this study, TYLCV DNA concentration in roots showed a belated increase, which demonstrated that a period of time is required for systemic transmission of TYLCV within tomato plants. Thus, a threshold period is needed for transmission of injury messages within the plant and for producing the defense compounds responsible for defense (Sauge et al., 2002). The acquisition of TYLCV by whitefly from TYLCV-infected tomato plants also displayed a tendency to rise and then decline. When increasing duration of aphid infestation beyond the 12 h threshold, the TYLCV concentration in whiteflies began to decrease. Aphids feeding on tomato plants for sufficient time can induce a plant defense response which inhibits whiteflies from acquiring TYLCV. Our result indicated that extension of the pre-infestation phase may increase virus transmission and acquisition by whitefly in the short term. This result might be explained by feeding efficacy, as influenced by the plant induced defense response.

### Feeding Behavior of *Bemisia tabaci* According to Aphid Pre-infestation

As our results show, the feeding behavior strategies of either TYLCV infected *B. tabaci* on non-infected tomato (virus transmission) or non-infected whitefly on viruliferous plants (virus acquisition) were both influenced by duration of pre-infestation by *M. persicae*. We found that the duration of phloem ingestion (E2) of *B. tabaci* may increase at 24 h and decrease at 48 h, with increasing of duration of pre-infestation. However, the total probing duration for whitefly was inconsistent between transmission/acquisition treatments. The transmission and acquisition of virus between whitefly and tomato plant may be due to different feeding strategies; for transmission of virus from whitefly to plant, net virus infestation is associated with salivation (E1). Thus, as indicated by the amount of TYLCV DNA in the host plant (**Figure 2**), virus infection in tomato may have a corresponding increase with salivation by viruliferous *B. tabaci*, although unaffected by total duration of probing. In contrast, the acquisition of virus by whitefly from infected plants was mainly related to phloem ingestion (E2). Clearly, our results showed similar responses to variation in aphid pre-infestation between the amount of TYLCV in whitefly and the duration of their phloem ingestion (**Figures 3, 4**). It seems that virus transmission/acquisition is influenced by the whitefly’s different feeding strategies. The feeding of whiteflies may be inhibited by the plant defense response triggered by aphid pre-infestation. Empirical reports indicate that defensive materials (like JA, SA and its derivants) may suppress phloem ingestion (Kehr, 2006). Thus, the non-viruliferous whitefly showed a reduced ability to combat this response, and struggle to maintain normal phloem ingestion feeding time. Interestingly, the virus infection may be quite specific in the way it regulates feeding behavior, with the whitefly enhancing only key processes, particularly salivation, which is vital for virus transmission.

### Gene Expression Related to SA- and JA-Signaling Pathways

Defense response to herbivore or plant pathogens occurs mostly in the Salicylic Acid (SA) and Jasmonic Acid (JA) signaling pathways (Lamb et al., 1989). Feeding by aphids induce plant responses similar to those induced by pathogens (Czosnek et al., 2002; Thompson and Goggin, 2006). Aphid infestation may activate the SA-signaling pathway and suppress the JA-signaling pathway, which will attract whiteflies and suppress TYLCV (Verhage et al., 2010). SA activation and JA suppression both inhibit viruses and improve feeding behavior of herbivore insects, respectively (Zarate et al., 2007). Our results showed that extending the period of aphid pre-infestation may reduce expression of the genes involved in the JA-signaling pathway, and increase expression of the genes involved in the SA-signaling pathway. According to our results, it seems that upregulating the SA pathway and suppressing the JA pathway with aphid pre-infestation may directly impact the feeding strategies of the whitefly, and consequentially, the transmission and acquisition of TYLCV between whitefly and tomato plant. Similarly, there have been several reports showing that induced plant defense responses are stronger against pathogens compared to insect herbivores (Ghanim et al., 2001; Tan and Liu, 2014). Ohgushi (2005) reported that infestation by tobacco mosaic virus can induce systemic acquired resistance, while not activating the JA signaling pathway. Generally, the SA pathway is activated by pathogens, and the JA pathway is induced by insects (Salzman et al., 2005). Previous studies have shown that attack by sap-sucking herbivores can induce plant responses similar to those induced by pathogen infestations, inhibiting the JA-signaling pathways and consequentially reducing negative impacts on herbivores (Papayiannis et al., 2009, 2010; Giordanengo et al., 2010). Plant defense responses induced by herbivores or pathogens are often similar, since both responses can act to resist both insects and pathogens, or to minimize interference between herbivores and pathogen defenses (Thaler et al., 2002; Murugan and Dhandapani, 2007). Tomato plants infested by phloem-feeding insects (e.g., aphids) evoked JA- and SA-signaling pathways to co-regulate the expression of plant defense genes (Muniyappa et al., 2000). The antagonism between SA and JA impacts feeding behavior of whiteflies and viral propagation. In the transmission test, the SA and JA was dominant during the whole process. However, during the acquisition test, the JA-signaling pathway was the key factor affecting virus acquisition, and the feeding behavior of whiteflies dominated the whole process. SA activity may deter the transmission of pathogens, but inhibition of JA might attract herbivorous insects (Chu et al., 2007). The results showed that a decrease in JA combined with an increase in SA led to a net positive effect of aphid infestation on increased feeding of whiteflies, outweighing inhibition of TYLCV transmission.

Furthermore, the defense responses of these two pathways may vary with duration of injury (Preston et al., 1999; Nombela et al., 2009). Our results not only showed a temporal component to variation in associated gene expression, but also showed that indirect systemic responses (variation in amount of TYLCV across plant organs) also vary according to duration of pre-infestation. Generally, herbivore feeding leads to systemic defense in the plant, enacted in a concerted manner across tissues and organs (Polston et al., 1990; Pico et al., 1999). Thus, further evaluation of defense-related gene expression induced by insect feeding on different plant parts is required, and how this is impacted by pre-infestation.

Generally, we have confirmed the hypothesis that aphid pre-infestation impacts transmission and acquisition of TYLCV between tomato and *B. tabaci*. Pre-infestation showed different respective influences to the feeding strategies during the transmission or acquisition processes, while associated with opposing trends in expression of the SA- and JA- pathways. Based on current understanding of the interactions between the SA- and JA- pathways, particularly cross-inhibition, we believe that the mechanism delineating the complex and precise interaction among these organisms is worthy of further research. Notwithstanding, due to the economic importance of tomato crop, our study might have implications in the development of novel strategies for the regulation of plant pathogens and insect vectors.

## Author Contributions

XT, JC, TL, and FG designed the study. All authors drafted the manuscript. XT, GB, ND, and XY implemented the analyses. All authors gave final approval for publication.

## Conflict of Interest Statement

The authors declare that the research was conducted in the absence of any commercial or financial relationships that could be construed as a potential conflict of interest.
